# Technological advances in the serological diagnosis of Chagas disease in dogs and cats: a systematic review

**DOI:** 10.1186/s13071-022-05476-4

**Published:** 2022-09-27

**Authors:** Natália Erdens Maron Freitas, Fernanda Lopes Habib, Emily Ferreira Santos, Ângelo Antônio Oliveira Silva, Natália Dantas Fontes, Leonardo Maia Leony, Daniel Dias Sampaio, Marcio Cerqueira de Almeida, Filipe Dantas-Torres, Fred Luciano Neves Santos

**Affiliations:** 1 Advanced Health Public Laboratory, Gonçalo Moniz Institute, Oswaldo Cruz Foundation, Waldemar Falcão Street, 121, Candeal, Bahia, Salvador 40296-710 Brazil; 2 Brazil’s Family Health Strategy, Municipal Health Department, Tremedal City Hall, Bahia, Tremedal Brazil; 3 Pathology and Molecular Biology Laboratory, Gonçalo Moniz Institute, Oswaldo Cruz Foundation, Salvador, Bahia Brazil; 4 Laboratory of Immunoparasitology, Aggeu Magalhães Institute, Oswaldo Cruz Foundation, Recife, Pernambuco Brazil; 5 Integrated Translational Program in Chagas Disease From Fiocruz (Fio-Chagas), Oswaldo Cruz Foundation, Rio de Janeiro, Brazil

**Keywords:** *Trypanosoma cruzi*, Dogs, Cats, Diagnosis, Serology, Epidemiology

## Abstract

**Background:**

Chagas disease (CD) is caused by *Trypanosoma cruzi*, which is transmitted mainly through the feces/urine of infected triatomine bugs. The acute phase lasts 2–3 months and is characterized by high parasitemia and nonspecific symptoms, whereas the lifelong chronic phase features symptoms affecting the heart and/or digestive tract occurring in 30–40% of infected individuals. As in humans, cardiac abnormalities are observed in *T. cruzi*-infected dogs and cats. We reviewed the technological advances in the serological diagnosis of CD in dogs and cats.

**Methods:**

A review of the published literature during the last 54 years (1968–2022) on the epidemiology, clinical features, diagnosis, treatment and prevention of CD in dogs and cats was conducted.

**Results:**

Using predefined eligibility criteria for a search of the published literature, we retrieved and screened 436 publications. Of these, 84 original studies were considered for inclusion in this review. Dogs and cats are considered as sentinels, potentially indicating an active *T. cruzi* transmission and thus the risk for human infection. Although dogs and cats are reputed to be important for maintaining the *T. cruzi* domestic transmission cycle, there are no commercial tests to detect past or active infections in these animals. Most published research on CD in dogs and cats have used in-house serological tests prepared with native and/or full-length recombinant antigens, resulting in variable diagnostic performance. In recent years, chimeric antigens have been used to improve the diagnosis of chronic CD in humans with encouraging results. Some of them have high performance values (> 95%) and extremely low cross-reactivity rates for *Leishmania* spp., especially the antigens IBMP-8.1 to IBMP-8.4. The diagnostic performance of IBMP antigens was also investigated in dogs, showing high diagnostic performance with negligible cross-reactivity with anti-*Leishmania infantum* antibodies.

**Conclusions:**

The development of a commercial immunodiagnostic tool to identify past or active *T. cruzi* infections in dogs and cats is urgently needed. The use of chimeric recombinant *T. cruzi* antigens may help to fill this gap and is discussed in this review.

**Graphical Abstract:**

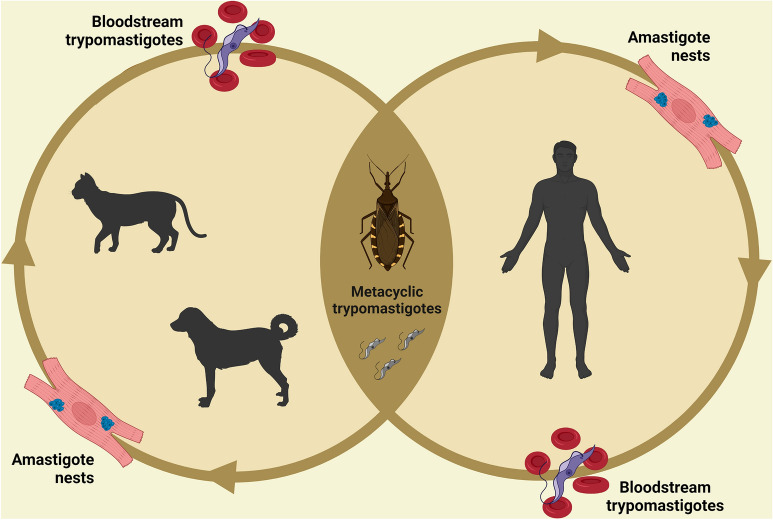

**Supplementary Information:**

The online version contains supplementary material available at 10.1186/s13071-022-05476-4.

## Background

**Fig. 1:**
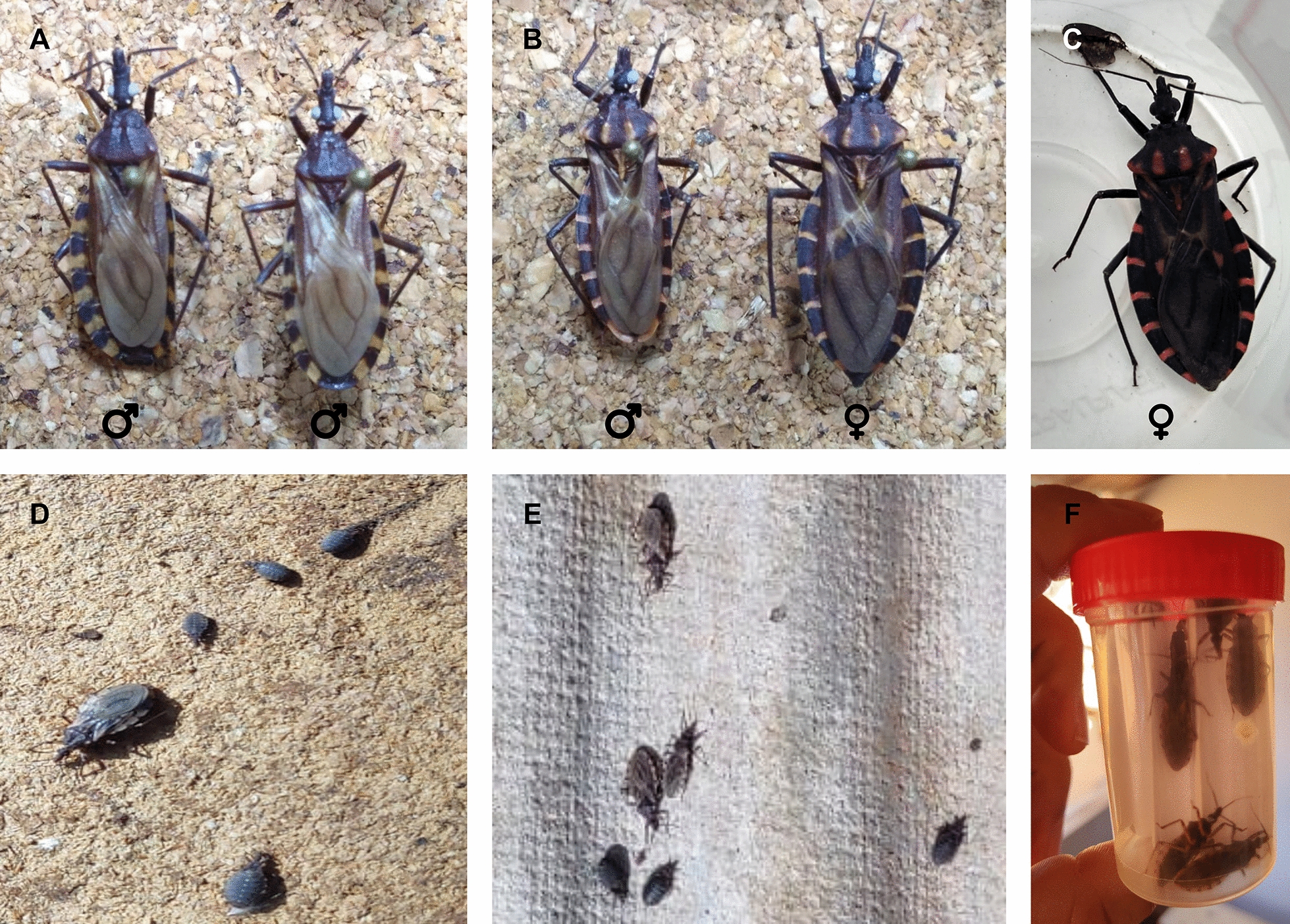
Triatomines normally found in endemic areas of South America. Preserved pair of *Triatoma infestans* **A** and *Panstrongylus megistus* **B**, kindly provided by Dr. Gilmar Jose da Silva Ribeiro Júnior (Fiocruz-Bahia). **C** shows a live *Panstrongylus megistus* female captured in the city of Barra do Mendes, Bahia, Brazil. Live triatomines of the species *Triatoma sordida* found on the floor **D** and roof **E** of a chicken house in the rural area of the municipality of Tremedal, Bahia. **E** shows some *T. sordida* adult specimens captured for gut content analysis

Chagas disease (CD) or American trypanosomiasis is a neglected parasitic disease caused by the hemoflagellate protozoan *Trypanosoma cruzi*. Recent estimates indicate that 6–7 million people are infected worldwide, with 10,000 deaths attributable to CD annually in 21 Latin American countries [1]. Due to the continuous presence of the vector, 70 million people in this region are at risk of contracting the disease via vector transmission [1]. The parasite is primarily transmitted through the feces or urine of infected bloodsucking triatomine bugs also referred to as kissing bugs (Hemiptera: Reduviidae) [1]. Over 130 triatomine species have been identified as potential vectors of *T. cruzi* [2]. Fifty-two triatomine species have been described in Brazil, of which five are considered of epidemiological importance because of their domestic habitats: *Triatoma infestans* (Fig. 1A), *Panstrongylus megistus* (Fig. 1B, C), *Triatoma brasiliensis*, *Triatoma pseudomaculata* and *Triatoma sordida* (Fig. 1D, E, F). Non-vectorial routes of transmission are also important for *T. cruzi* transmission, such as blood transfusion or the use of blood products, congenital transmission, consumption of contaminated food and beverages, organ donation and laboratory accidents [3]. Increased travel and migration flows have facilitated the spread of *T. cruzi*-infected individuals, making the disease a global health problem, particularly in non-endemic countries in Europe, North America, Asia and Oceania [4–7].

Clinically, human CD is divided into two phases: acute and chronic. The acute phase begins within 1–2 weeks of infection, lasts 2–3 months and is characterized by high parasitemia and nonspecific symptoms such as fever, tachycardia and lymphadenopathy [8]. The lifelong chronic phase can occur in two forms: an indeterminate form, which is usually a latency period in which individuals show no symptoms but have positive serological results, and a symptomatic form. Approximately 30–40% of chronically infected individuals progress to a symptomatic form, which can be further subdivided into cardiac, digestive, or mixed forms (cardiac and digestive) [8]. As in humans, cardiac disease is also observed in *T. cruzi*-infected dogs [9–11] and cats [12]. Indeed, some animals develop progressive chronic myocarditis with cardiac dilatation and electrocardiogram abnormalities that may lead to sudden death. Clinical signs may include splenomegaly, lymphadenopathy and heart failure [13].

**Fig. 2:**
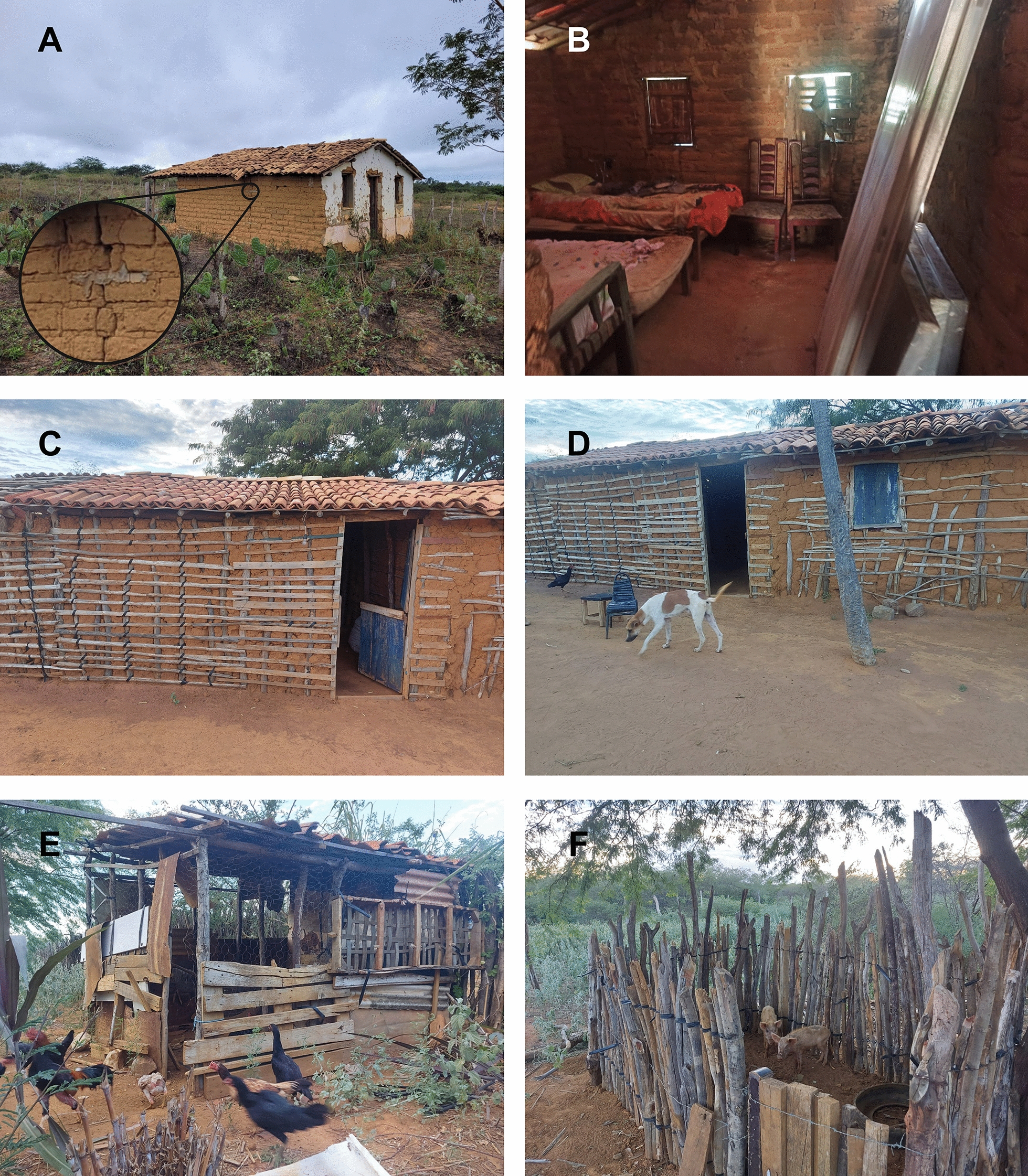
The epidemiologic scenario of poor rural communities in many Latin American countries. Mud house with cracks where triatomines can hide (**A**-**C**), with detail of a crack in a rural adobe/brick house (**A**). **B** Inside of the house illustrated in (**A**). Mud house with cracks (**C**) with presence of domestic animals in the environment: dog (**D**), chickens (**E**) and pigs (**F**). These animals can attract triatomines for a blood meal, thus helping maintain the peridomestic cycle of *T. cruzi*. Photographs were taken in rural areas of the municipalities of Tremedal (**A** and **B**) and Irecê, Bahia, Brazil (**E**–**F**)

Although CD was discovered more than a century ago, this zoonosis still poses a public health threat [14]. The presence of domestic animals in the environment is a risk factor for human infection because they may attract triatomines to human dwellings. Indeed, triatomines typically feed on chickens, pigs, dogs and cats [15, 16]. Figure 2 shows a typical scenario in poor rural communities in Latin American countries where mud houses are still common (Fig. 2A, E, F) and domestic animals such as dogs, chickens and pigs are present (Fig. 2B, C, D respectively). Among domestic animals, dogs and cats play an important role in maintaining the domestic cycle of *T. cruzi*, since these animals are susceptible to different forms of infection [17, 18]. They are reputed to be the main reservoirs of *T. cruzi* in Latin America and some regions of the US [13, 19–21]. Dogs and cats are also considered as sentinels for human infection [22, 23], since they can indicate the presence of an active *T. cruzi* transmission cycle and thus the risk of human infection.

Despite the public health and veterinary importance, there are no commercially available tests to detect past or active *T. cruzi* infections in dogs and cats. In this review, we summarize basic information on CD in dogs and cats, with particular emphasis on diagnostic methods.

### Search strategy, eligibility and review

An online search was performed in the US National Library of Medicine National Institutes of Health (PubMed, Bethesda MD, USA; https://pubmed.ncbi.nlm.nih.gov/), the Latin American and Caribbean Health Science Literature Database (LILACS; https://lilacs.bvsalud.org/) and the Scientific Electronic Library Online (Scielo Brazil, São Paulo SP—Brazil; https://scileo.br/) databases using Health Sciences Descriptors (DeCS). The descriptors in the different databases were “Chagas disease,” “dogs,” and “cats” in Portuguese, English and Spanish. The Boolean operator “AND” was used to cross descriptors and keywords. Search results were then filtered for the period 1968 to 2022 and extracted into a database in Microsoft Excel (Microsoft Corp., Redmond, WA, USA) in CSV format (comma-separated values).

Inclusion criteria were (1) articles indexed in the previously cited databases; (2) original studies in Portuguese, Spanish, or English; (3) published between 1968 and 2022. The survey took place from January to June 2022, excluding secondary publications such as books, monographs, dissertations and theses.

**Fig. 3:**
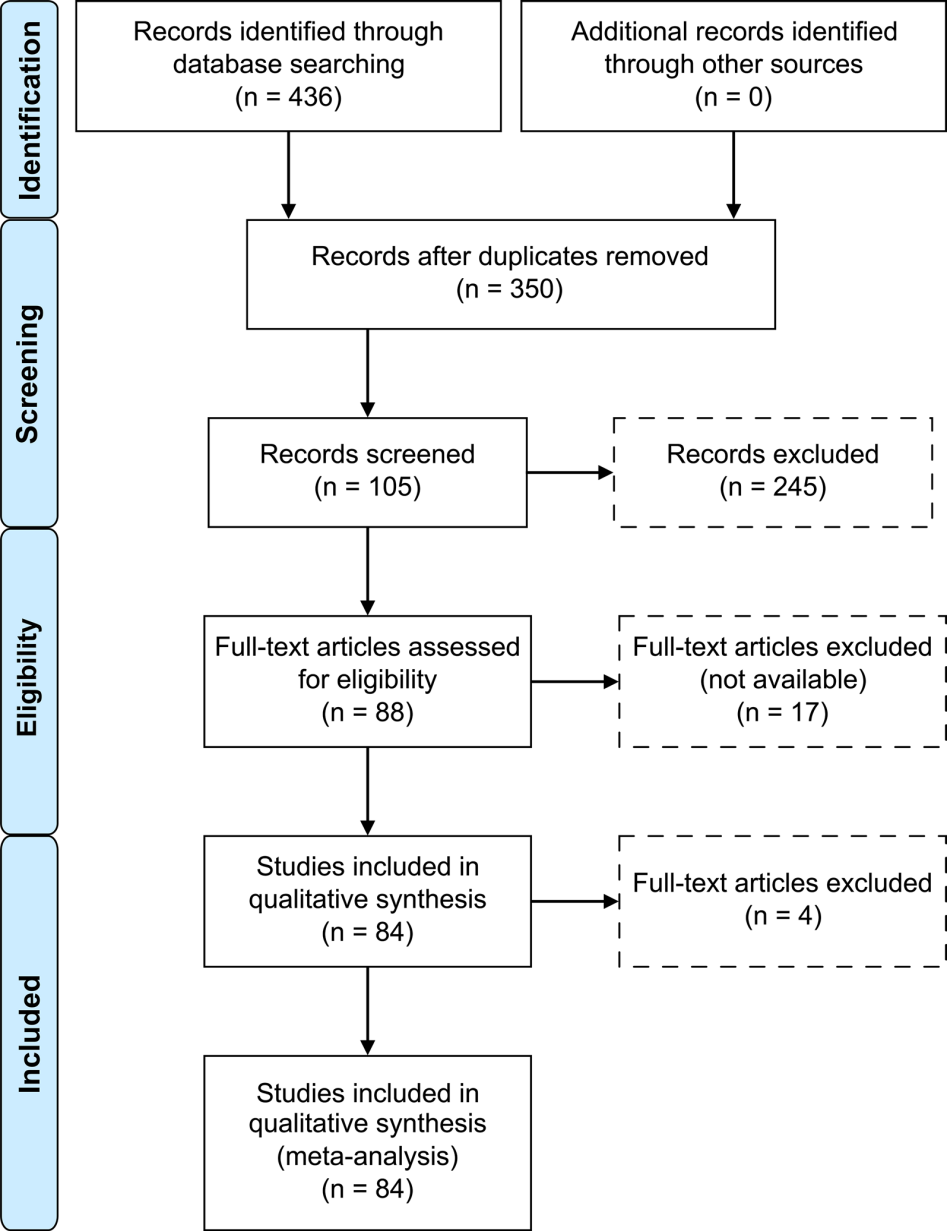
Study selection process, in accordance with the PRISMA model

The extraction and analysis of primary data were conducted by two independent researchers. Exclusion of articles was based first on reading the titles as the first analysis, followed by reading the abstracts (if available) and finally reading the full texts. In case of doubts or discrepancies, a third researcher was consulted. A total of 436 articles were found during the initial search. After an initial analysis of the titles, 191 articles were excluded, and another 86 articles were not included because they were duplicates. Of the remaining 159 articles, the abstracts were read and 106 were considered for conducting the integrative review. Of these, 17 articles were excluded because they were not available in the scientific literature. After the qualitative analysis, 84 of the 89 selected articles were considered for conducting this review. The process of study selection was performed according to the Preferred Reporting Items for Systematic Reviews and Meta-Analyses (Additional file 1: PRISMA) model [24], which is shown in Fig. 3.

### *Trypanosoma cruzi* infection and CD in dogs

**Fig. 4:**
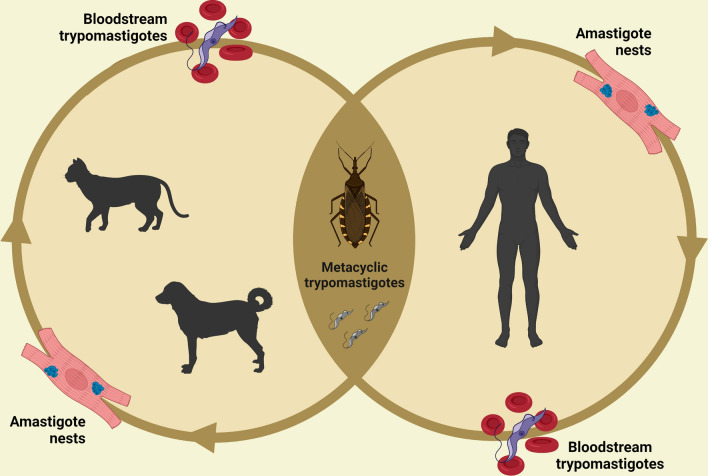
Developmental stages of *Trypanosoma cruzi* in invertebrate (triatomine bug) and vertebrate (dog, cat and human) hosts

Dogs become infected after coming in contact with the feces of triatomine bugs containing *T. cruzi* trypomastigote (Fig. 4), by ingesting infected triatomines, or congenitally [25]. They are considered the most important domestic reservoirs of *T. cruzi* in areas where CD is endemic owing to their proximity to humans, high parasitemia in acute phase of the disease and propensity to attract triatomines [17, 20, 22]. Nonetheless, the role of dogs as reservoirs may apparently vary. For instance, a study conducted in Brazil showed that dogs from some regions presented negative blood cultures and fresh blood preparations [26], whereas studies conducted in Argentina [22, 27] and Brazil [28] indicated that vast majority of seropositive dogs had active parasitemia.

A study conducted in 1996 estimated that dogs contribute 13.9 times more than humans to triatomine infection in households [29]. Accordingly, the likelihood of a triatomine bug to become infected by *T. cruzi* was found 50 times higher after a single blood meal on a dog than on a human [29]. In another study examining the feeding habits of over 1000 domestic *T. infestans*, it was found that dogs were the most common blood source (49%), followed by cats (39%), humans (38%) and chickens (29%) [17].

**Fig. 5:**
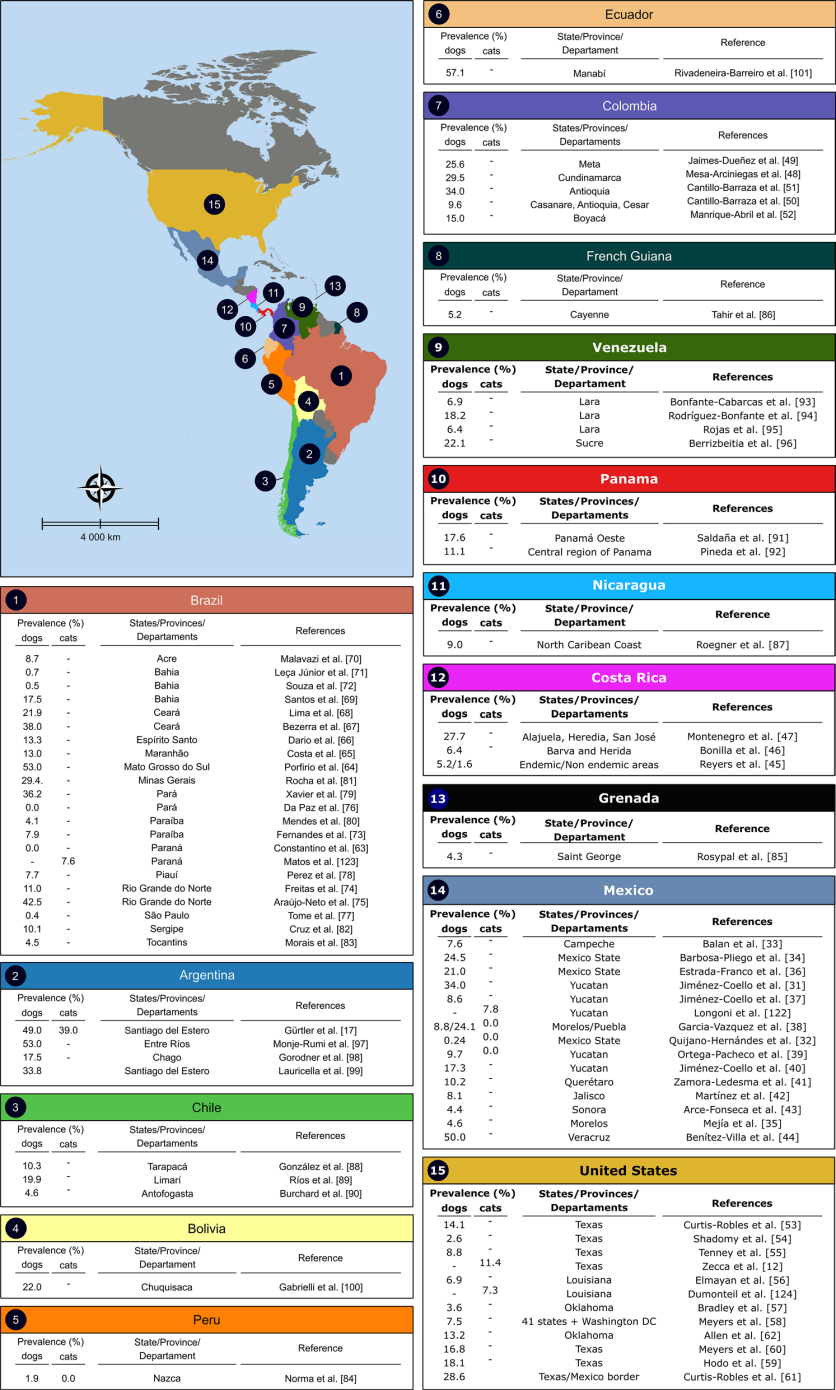
Seroprevalence of Chagas disease in dogs and cats in different endemic countries of North, Central and South America

Considering the role of dogs as reservoirs for *T. cruzi*, the seroprevalence in dogs has also been used in mathematical models for domestic transmission of human CD [30]. However, seroprevalence data from different countries may vary widely (Fig. 5), which may be partly attributed to the variability in terms of transmission risk, but also to sample size and serological tests used by different research groups to detect anti-*T. cruzi* antibodies in dogs, as well as to the genetic variability of the circulating *T. cruzi* strains (discrete typing units (DTU). In Mexico, a study conducted in 2010 reported a prevalence of 34% [31], while another study carried out in 2017 detected a prevalence of 4.4% [32]. Other studies conducted in Mexico have reported different prevalence values [33–43]. Recently, anti‑*T. cruzi* antibodies were detected in 50% (17/34) of dogs from two rural settlements in the Sierra de Los Tuxtlas, Veracruz, Mexico [44]. In Costa Rica, 5.2–27.7% of dogs were seropositive in endemic areas [45–47], whereas in Colombia the prevalence ranged from 9.6% to 34% [48–52]. In the US, a study with 86 working dogs reported a seroprevalence of 14.1% [53], whereas other studies reported a lower prevalence [54–57]. *Trypanosoma cruzi* infection was reported in 63 (16.8%) of 375 dogs from a teaching hospital in Texas [58] and in 110 (18.1%) of 608 dogs in shelters across this same state [59]. In 2020, an American nationwide study using dog samples from 41 states and Washington DC revealed a seropositivity in 120/1610 animals (7.5%) [60]. In a National Park located along the Texas-Mexico border, 28.6% (4/14) of dogs were reactive on at least two serologic assays [61]. More recently, 26 of 197 (13.2%) shelter dogs from Oklahoma had detectable antibodies against *T. cruzi* [62]. In Brazil, the seroprevalence in dogs ranged from 0 to 53%, according to research conducted in different regions [63–83]. In other Latin American countries, the seroprevalence varies according to geographic setting, e.g. 1.9% in Peru [84], 4.3% in Grenada [85], 5.2% in French Guiana [86], 10% in Nicaragua [87], 4.6–19.9% in Chile [88–90], 11.1–17.6% in Panama [91, 92], 6.4–22.1% in Venezuela [93–96], 17.5–53% in Argentina [17, 97–99], 22% in Bolivia [100] and 57.1% in Ecuador [101].

Regarding predisposing factors, it has been observed that dogs with poor nutritional conditions are 6.3 times more likely to be infected compared to well-nourished dogs in the same endemic area [102]. This is thought to be related to a deficient innate immune response in dogs with poor nutritional conditions, which favors the occurrence of higher parasitemia [102]. Another predisposing factor is keeping dogs in kennels with multiple dogs. In fact, an American study found that the risk of *T. cruzi* infection in dogs living in kennels is 30.7% per year [103].

**Fig. 6:**
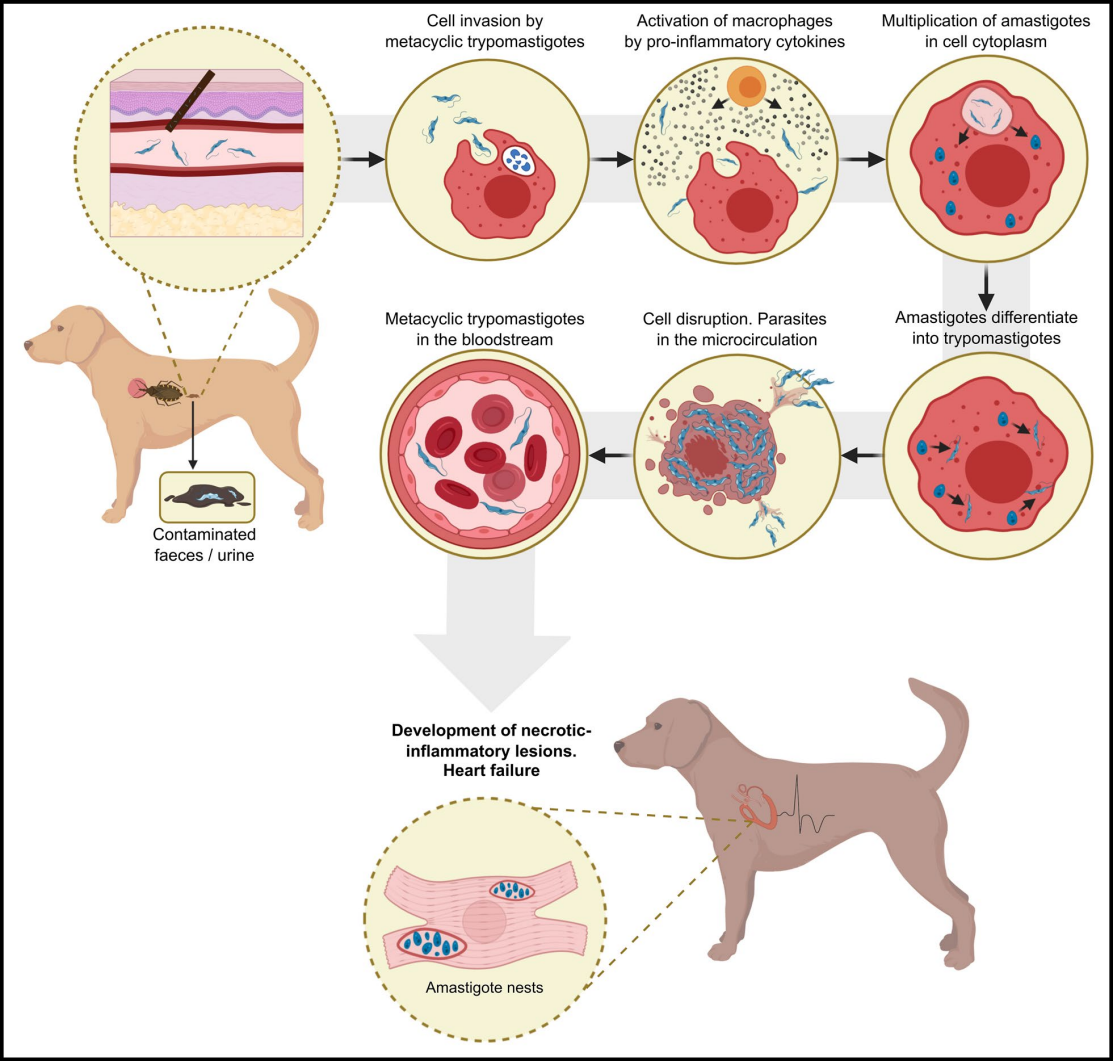
Schematic representation of the pathogenesis of Chagas disease in *Trypanosoma cruzi*-infected dogs

During the acute phase of infection, *T. cruzi* circulates in the bloodstream and trypomastigotes can be observed in most tissues, triggering a systemic inflammatory response with the production of proinflammatory cytokines (Fig. 6).

Clinical signs vary widely according to infection phase (acute versus chronic) and to dog’s age. For instance, the main presenting clinical signs in young puppies are lethargy, generalized lymphadenopathy, slow capillary refill time with pale mucous membranes and in some cases splenomegaly and hepatomegaly [13]. On the other hand, if infection occurs after 6 months of age, dogs may display no signs of acute disease other than slight depression and low-rising parasitemia [13]. In general, the main lesion in young dogs experimentally infected with *T. cruzi* is acute myocarditis that begins in the atria and spreads through the interventricular septum toward the ventricles [104]. When fully developed, it is located predominantly in the right atrium, the right half of the ventricular septum and the free wall of the right ventricle. Electrocardiogram (ECG) changes are progressive and reflect atrial involvement. Heart block occurs only in the terminal stage and is associated with severe inflammation and necrosis along the A-V conduction tissue. Specific treatment of dogs with severe acute disease often results in regression of histologic and ECG changes [104].

Dogs that survive the acute phase enter the undetermined phase, characterized by the lack of clinical signs and subpatent parasitemia [13]. Some dogs will progress to develop chronic disease, typified by cardiac alterations, including chronic myocarditis with cardiac dilatation [13, 105, 106]. During this phase electrocardiogram abnormalities become more evident [13, 107, 108]. Other lesions are associated with fibrosis and cardiomyocyte necrosis, possibly caused by the inflammatory processes that trigger hyalinization and fibrosis [107–110]. Right-side and, eventually, left-side chamber failure may occur, inducing pulse deficits, ascites, pleural effusion, hepatomegaly and jugular venous congestion [13, 111, 112]. In general, naturally infected dogs showed hyperproteinemia, low hemoglobin and hematocrit levels, hypoalbuminemia, hyperglobulinemia, high lactate dehydrogenase (LDH) and aspartate transferase (AST) levels, creatine kinase (CK) and creatine kinase myocardial band (CK-MB) and troponin I profiles consistent with active myocarditis [113–116].

Chagasic megaesophagus and megacolon can be observed in both the acute and chronic phases [117]. In the acute phase, *T. cruzi* triggers an inflammatory reaction in the esophagus/colon and causes myenteric denervation. Ganglionitis and periganglionitis of the Auerbach's plexus ranged from mild to moderate and resulted in significant neuronal lesions in dogs experimentally infected with *T. cruzi* strain Berenice-78 [118]. In the chronic phase, persistent myenteric denervation occurs and may lead to impaired digestive function. Glial cell involvement occurs in the acute phase and may lead to a decrease in the glial fibrillary acidic protein immunoreactive area of enteric glial cells in the chronic phase [117].

Prognosis may be unpredictable and the survival rate of chronically infected, untreated dogs is variable. For instance, dogs diagnosed with CD at an older age tend to survive longer than dogs diagnosed at a younger age [13]. A study showed that a combination of amiodarone and itraconazole may increase the survival time of *T. cruzi*-infected dogs [119]. On the other hand, a recent study showed that two dogs with severe, symptomatic Chagas cardiomyopathy treated with itraconazole and amiodarone died suddenly within 6 months of diagnosis [120]. These findings underscore the need for early recognition of CD in dogs and continued research to develop effective antiparasitic treatment protocols.

### *Trypanosoma cruzi* infection and CD in cats

Chagas disease in cats is not as well studied as in dogs. Risk factors for the development of CD in cats are still unclear, but it appears that free-roaming cats are more susceptible to *T. cruzi* infection and are an important risk factor for transmission to humans [37]. Indeed, xenodiagnosis data indicated that cats are highly likely to infect peridomestic triatomine vectors [17]. Clinical signs and histologic findings in cats are similar to those described in humans and dogs [121]. Although more common in humans, digestive symptoms such as esophagitis have also been described in *T. cruzi*-infected cats [12]. Neurological signs associated with CD have been described in several species, including dogs [13], but never in cats.

Some cats can mount an effective immune response to *T. cruzi*. However, when immunocompromised or coinfected with other infectious agents [such as feline infectious peritonitis, feline leukemia virus (FELV), feline immunodeficiency virus (FIV) and feline herpesvirus type 1], they may be more susceptible to *T. cruzi* [37], but this is something that requires further study.

There are few studies on the epidemiology of *T. cruzi* infection in cats (Fig. 5). In a study conducted in three Mexican cities (Mérida, Umán and Tulum) in Yucatán, 7.8% of 95 cats were positive by ELISA and Western blot, using excreted superoxide dismutase as antigen [122]. Interestingly, no infection was observed in other studies conducted in distinct parts of Mexico [32, 38, 39].

A study conducted in Paraná (Brazil) showed 30.8% of 679 cats had anti-*T. cruzi* antibodies detectable by IFAT and 23.6% by ELISA. Only 7.6% of the cats were simultaneously positive to both tests, showing a large discrepancy between these methods [123]. Recent studies in the US revealed 7.3–11.4% of the cats were seropositive in South Texas [12] and Louisiana [124], respectively. Among studies that investigated *T. cruzi* infections in cats, the highest prevalence was reported in Trinidad and Mercedes, two rural villages in the province of Santiago del Estero, Argentina [17]. The authors found an overall seroprevalence of 39–40% in cats at baseline and 1 year later, respectively. Seroprevalence was found to increase with age but was not with sex [17].

### Diagnostic methods

**Fig. 7:**
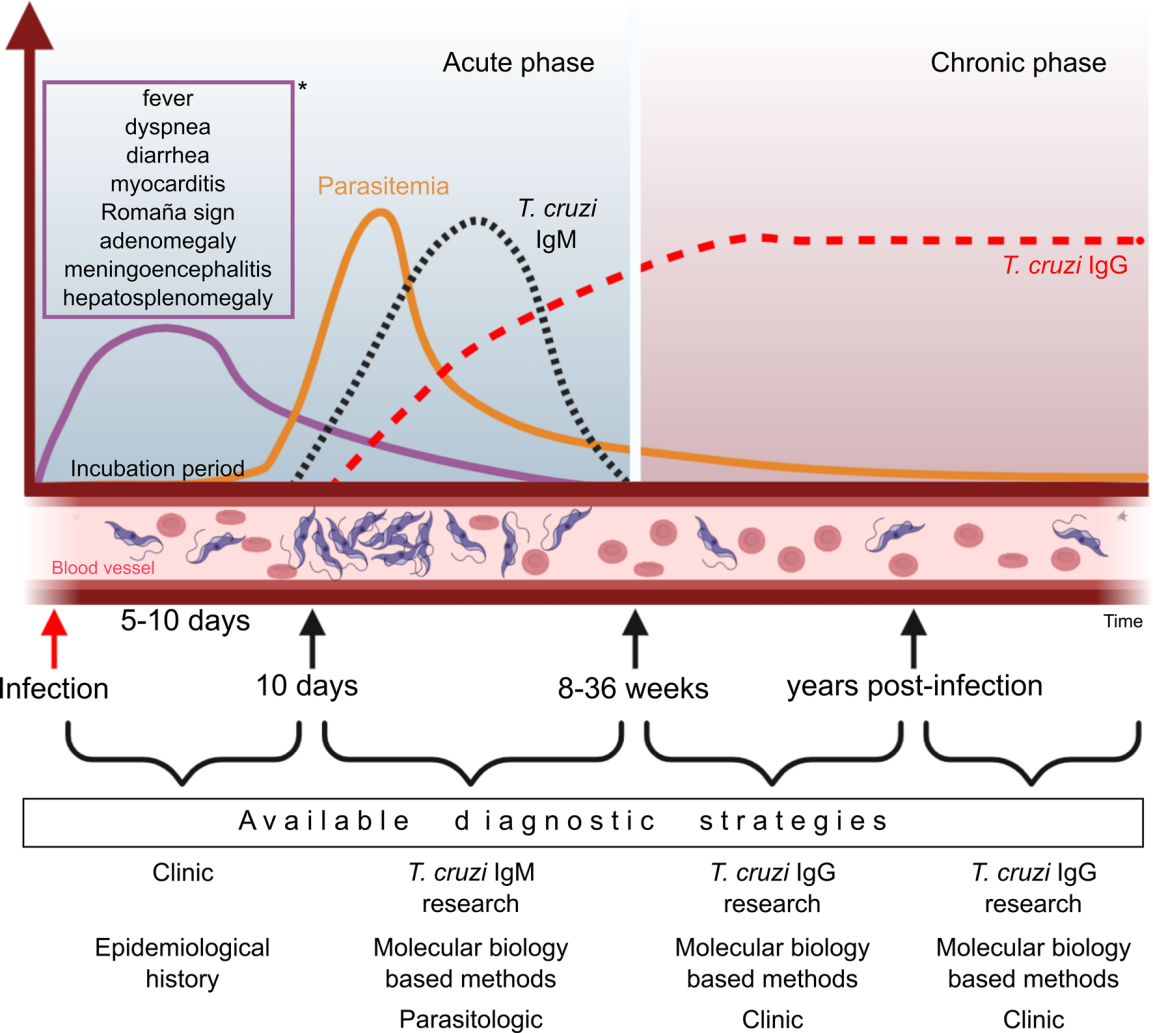
Schematic representation of the natural history of *Trypanosoma cruzi* infection in dogs. *In the acute phase, most infected animals are asymptomatic, but when symptomatic, they may present the described clinical signs

In endemic areas, the presence of the above-mentioned clinical signs and clinicopathological abnormalities can lead veterinarians to suspect CD in dogs and cats. In the acute phase, parasitological (e.g., fresh or stained blood preparations, hemoculture and xenodiagnosis) [26] or molecular methods [125] may be useful to confirm the infection (Fig. 7). In addition, molecular methods may be useful for monitoring parasitemia during drug treatment of CD in dogs [126]. However, parasitemia in dogs and cats is generally low and intermittent in the chronic phase [106], which reduces the sensitivity of parasitological and molecular methods. On the other hand, during the chronic phase, anti-*T. cruzi* antibody production reaches detectable titers and can be identified by indirect immunoassays. In dogs, serum immunoglobulin M (IgM) begins to decrease markedly about 3 months after infection, whereas the opposite is true for immunoglobulin G (IgG), which increases up to 15 months and then gradually decreases up to 2 years and then appears to stabilize over the years [127].

Table 1 summarizes serological methods and antigens previously used in studies involving dogs and cats. Some of the tests described were manufactured for the diagnosis of CD in humans, but they have been adapted for dogs and cats. One of the major drawbacks of this adaptation is the lack of phase 1 and phase 2 studies to validate the method in dogs and cats, so the results may not be reproducible. For example, Zecca et al. (2020) used two immunochromatographic tests developed for humans—Chagas Stat-Pak (Chembio Diagnostic Systems, Inc., Medford, NY) and Chagas Detect Plus Rapid Test (InBios International, Inc., Seattle, WA)—to detect anti-*T. cruzi* antibodies in cats. Although both tests use protein A to detect IgG antibodies, there are no studies validating their use in cats.

**Table 1:** Serological tests and antigens used in studies with dogs and cats

Method	Antigen or manufacturer	Performance assessment?	Sample and sample size	References
EIA	Whole lysate extracted from epimastigotes of Colombian *T. cruzi* strains Cas-15 and Gal-61	No	Serum D (*n* = 251)	[178]
IFAT	Complete epimastigotes of Colombian *T. cruzi* strains Cas-15 and Gal-61			
IHA	Chagastest HAI test (Wiener Laboratories Rosario, Argentina)			
EIA	TSSApep lineage-specifc	No	Serum D (*n* = 85) C (*n* = 19) A (*n* = 7)	[179]
RDT	Chagas Sero K-SeT RDT			
EIA	Laboratorio-Lemos SRL, Buenos Aires, Argentina	No	Serum D (*n* = 291)	[32]
IHA	Polychaco, Laboratorio-Lemos SRL, Buenos Aires, Argentina			
IFAT	According to [180]—(Tulahuén strain)	No	Serum D (*n* = 54)	[47]
EIA	ELISA of Biozima kit (Polychaco)			
IHA	Hemacruzi (BioMerieux)			
IFAT	According to [180]—(Tulahuén strain)	No	Serum D (*n* = 182)	[181]
IHA	Polychaco S.A.I.C, Buenos Aires, Argentina			
EIA	Homogenate of the flagellar fraction of *T. cruzi*			
IFAT	Complete epimastigotes of F90 and Y88 strains	No	Serum D (*n* = 330)	[65]
EIA	Chimeric recombinant proteins IBMP			
RDT	InBios Stat-Pak rapid test	No	Serum C (*n* = 284)	[124]
EIA	*T. cruzi* parasite lysate from strain WB1			
RDT	Trypanosoma Detect2 MRA Rapid Test. Inbios International Ltd., Washington, USA	No	Serum D (*n* = 67)	[85]
EIA	Whole lysate extracted from epimastigotes of NC-9 strain	No	Serum D (*n* = 2)	[182]
IFAT	Complete epimastigotes of NC-9 strain			
IFAT	Complete epimastigotes of Panamanian Burunga strain	No	Serum D (*n* = 99)	[92]
EIA	ELISA Chagastest. Wiener Lab., Argentina modified with whole lysate extracted from epimastigotes of Panamanian Burunga strain			
EIA	Iron superoxide dismutase—FeSODe	No	Serum D (*n* = 303)	[41]
WB	Iron superoxide dismutase—FeSODe			
IFAT	According to [180]	Yes	Serum D (n = 481)	[183]
IHA	Polychaco SAIC, Buenos Aires, Argentina			
CFT	According to [184]			
DAT	Polychaco SAIC, Buenos Aires, Argentina			
IHA	Polychaco, Buenos Aires, Argentina	No	Serum D (*n* = 86) C (*n* = 38)	[17]
EIA	Homogenate of the flagellar fraction of *T. cruzi*			
IFAT	According to [180]—(Tulahuén strain)			
RDT	Trypanosoma Detect, Inbios, Washington, USA	Yes	Serum D (*n* = 199) C (*n* = 57)	[185]
IHA	Polychaco, Buenos Aires, Argentina			
EIA	ELISA A: anti-IgG-HRP; Santa Cruz Biotechnology, Santa Cruz, CA ELISA B: using recombinant trans-Sialidase as antigen			
IFAT	Fluorescein-conjugated anti-gammaglobulin LID; Laboratorio Inmunodiagnóstico, Buenos Aires, Argentina			
WB	TESA-blotting, BioMerieux based on strain Y	No	Serum D (*n* = 111)	[83]
RDT	Chagas StatPak^®^ Assay, Chembio, USA	No	Serum D (*n* = 153)	[86]
EIA	Total proteins from Querétaro strain of *T. cruzi*	No	Serum D (*n* = 209)	[42]
WB	Total proteins from Querétaro strain of *T. cruzi*			
IFAT	Complete epimastigotes of Colombian *T. cruzi* strains I00/BR/00F (TcI) and MHOM/BR/1957/Y (TcII)	No	Serum D (*n* = 62) WM (*n* = 36)	[64]
EIA	ELISA, Bio-Manguinhos, Rio de Janeiro, Brazil			
EIA	Plates of Chagas III ELISA kit (Grupo Bios^®^) and monoclonal secondary antibody goat anti-dog IgG-HRP: sc-2433 (Santa Cruz Biotechnology, INC)	No	Serum D (*n* = 356)	[48]
IFAT	N.I.H.—Colombia			
RDT	Chagas Sero K-SeT (TSSA peptide epitope specific to TcII/V/VI)	Yes	D (*n* = 57)	[172]
RIPA	Tulahuén strain epimastigote lysate	No	Serum D (n = 301)	[57]
RDT	Chagas Stat-Pak (Chembio, Medford, NY, USA)	No	Serum D (*n* = 540)	[56]
EIA	Whole parasite lysate from a local strain WB1			
WB	Whole parasite lysate from a local strain WB1			
RDT	Chagas Stat-Pak (Chembio, Medford, NY, USA) Chagas Detect Plus Rapid Test (InBios International, Inc., WA, USA)	No	Serum C (*n* = 167)	[12]
IFAT	Texas Veterinary Medical Diagnostic Laboratory (TVMDL, College Station, TX)			
EIA	Recombinant proteins PGR31-His, PGR30-His and PGR24-His	No	Serum D (*n* = 333)	[93]
MABA	Recombinant proteins PGR31-His, PGR30-His and PGR24-His			
EIA	Modified Gold ELISA Chagas commercial test Chimeric recombinant proteins IBMP	No	Serum Dogs (*n* = 40)	[69]

*A* (armadillos), *C* (cats), *CFT* (complement fixation test), *D* (dogs), *DAT* (direct agglutination test), *IBT* (immunoblot test), *IFAT* (immunofluorescence antibody test), *MABA* (microplate alamar blue assay), *RDT* (rapid diagnostic test/immunochromatography), *WB* (Western blot), *WM* (wild mammals) *TSSA* (mucin trypomastigote small surface antigen)

Whole-cell homogenates or fractionated lysates of *T. cruzi* epimastigotes have traditionally been used as complex antigen mixtures to detect anti-*T. cruzi* antibodies. Although these combinations have been shown to provide sufficient sensitivity to detect even low antibody levels [128], difficulties in standardization, cross-reactivity and specificity issues have hindered their use in humans [129–132]. This is especially true for IFAT and ELISA results used to diagnosis CD in humans and other species, which may vary depending on the circulating *T. cruzi* strain in the study area and the epimastigote strain used in the tests [65, 133, 134]. Another drawback is the use of different epimastigote strains in IFAT because *T. cruzi* has a high antigenic variation that can lead to false-negative or false-positive results [65], depending on the geographic region.

In the last 2 decades, advances in DNA recombination technology have enabled the use of recombinant proteins in immunoassays (primarily ELISA and chemiluminescence assays), as large quantities of purified antigens can be produced in transformed prokaryotic cells grown in bioreactors [135]. Approximately 25% of the proteins expressed by *T. cruzi* contain tandem repeat amino acid sequences consisting of 5–68 amino acids [136–139]. This improved recognition by antibodies compared to proteins that lack repeated sequences [130, 140, 141] and improved the performance of immunoassays compared to cell extracts or whole epimastigotes [136, 142]. Indeed, sera from infected humans often contain high titers of antibodies against these repeated sequences [143–145]. However, it has been observed that assays using recombinant proteins can also lead to false-negative results [130]. The high genetic variability of the parasite may be responsible for these results, as the tandem repeat amino acid sequences contain a limited repertoire of antigenic determinants that are not expressed or only partially expressed in some *T. cruzi* strains [146]. To overcome this limitation, several studies have described the combined use of two or more recombinant proteins in a single assay to increase sensitivity without losing specificity [147–151]. In theory, this strategy could compromise assay performance due to imbalanced binding of these epitopes to the solid surface, competition for binding and spatial distribution of epitopes in the solid phase. However, immunoassays containing a mixture of fusion proteins showed good performance [136]. More recently, an array of different antigens printed in each well of 96-well plates has been shown potentially useful for the diagnosis of human chronic CD [152].

In recent years, synthetic chimeric recombinant antigens consisting of conserved repetitive amino acid fragments of different antigenic *T. cruzi* proteins have been proposed to improve the accuracy of immunoassays for the diagnosis of human CD [130, 136, 153]. In 1999, a study investigated the diagnostic potential of a branched synthetic peptide (2/D/E/Lo1.2) and a linear recombinant peptide (r2/D/E/Lo1.2). The results showed that both antigens increased the reactivity of weakly reactive sera [137]. High diagnostic performance was obtained in a study examining antigens CP1, CP2 or a mixture between them. CP1 contains repetitive fragments of flagellar repetitive antigen (FRA) and shed acute phase antigen (SAPA), whereas CP2 consists of amino acid sequences of three antigens: FRA, SAPA and B13 [136]. The discriminative ability values obtained for CP1 and CP2 were 25% and 52% higher, respectively, than those of their individual antigen mixtures. CP2 was the only antigen that showed higher discriminative capability between *T. cruzi*-positive and -negative samples compared to the homogenate of the whole parasite [136]. Similar results were obtained with a chimeric antigen designated TcBCDE, a 24-kDa fusion protein composed of repetitive sequences of nine *T. cruzi* proteins (MAP, JL8, CRA, B13, TcD, TcE and SAPA). This antigen was evaluated and proven to be highly sensitive for the diagnosis of human CD [154]. A chimeric protein called CP3, composed of the antigenic determinants microtubule-associated protein (MAP), TcD and trypomastigote small surface antigen (TSSA)-II/V/VI, was 100% sensitive and 90.5% specific [155]. These results not only demonstrate that chimeric recombinant proteins are highly accurate in the diagnosis of chronic CD, but also that they are able to detect anti-*T. cruzi* antibodies regardless of parasite strain or gene expression intensity. In addition, these findings support the utility of performing immunochemical assays with hybrid, chimeric single-molecule antigens rather than peptide mixtures or recombinant proteins.

**Fig. 8:**
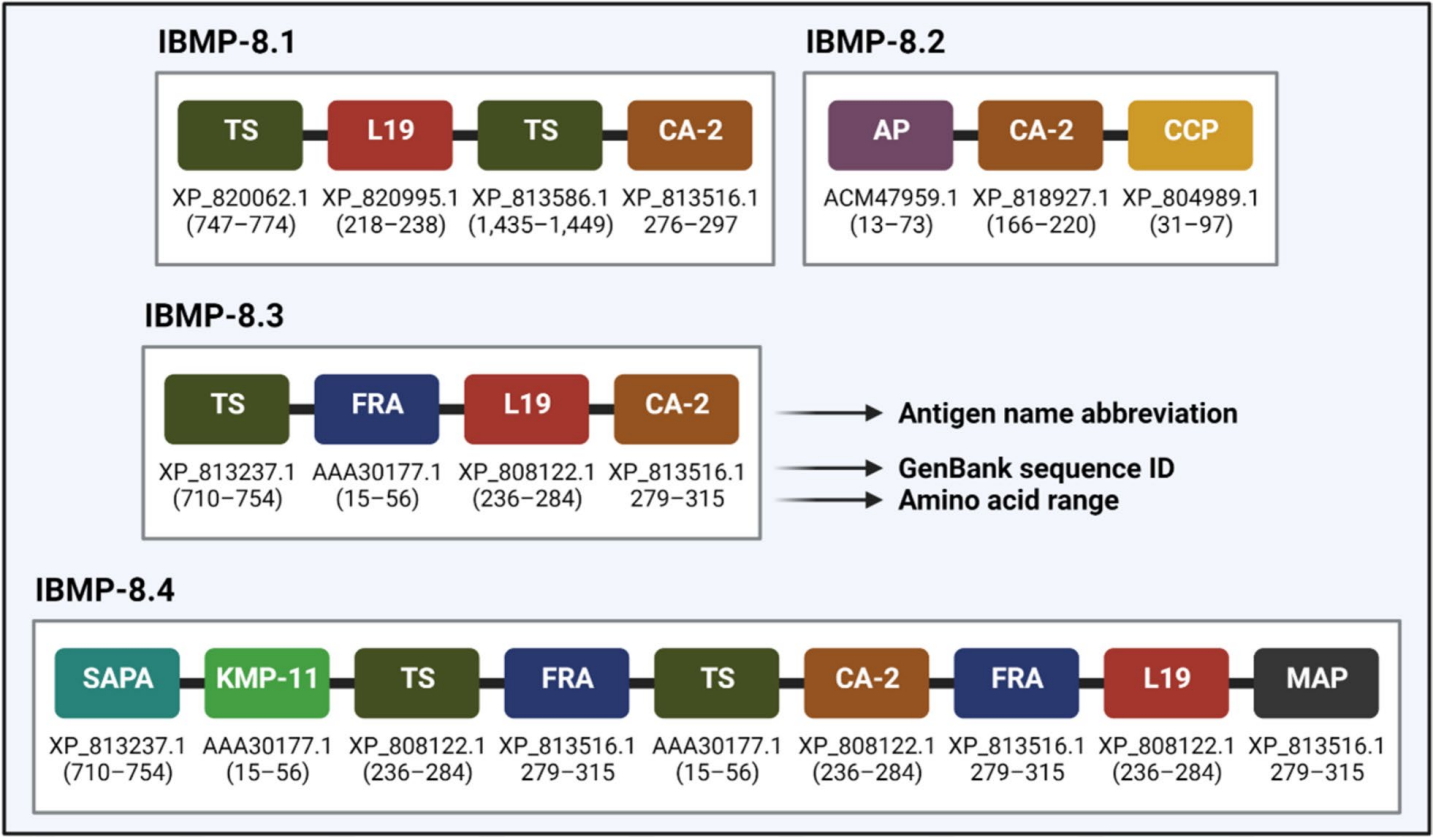
Constitution of the IBMP chimeric recombinant proteins [156, 159, 166]

Recently, four chimeric recombinant *T. cruzi* antigens have been proposed for the diagnosis of chronic CD in humans: IBMP-8.1, IBMP-8.2, IBMP-8.3 and IBMP-8.4 (IBMP is the Portuguese acronym for Biology Molecular Instituto of Paraná, where the antigens were expressed and purified). The diagnostic potential of these proteins for the detection of CD in humans has been extensively studied, using different diagnostic methods and platforms such as indirect ELISA [129, 156–161], liquid microarray [162], lateral flow assay [163], double-antigen sandwich ELISA [164], Western blot (unpublished data) and immunosensor [165]. IBMP antigens are composed of different epitopes of several *T. cruzi* proteins, as described in Fig. 8 [156, 159, 166]. In general, this diversity of antigenic determinants is responsible for their high reactivity to anti-*T. cruzi* antibodies.

The ability of IBMP antigens to discriminate *T. cruzi*-positive from -negative human samples was evaluated, and the area (AUC) under the receiver-operator curve was determined for each molecule. The determination of AUC values is used as the global accuracy of immunoassays [129] and can be classified as low (51–61%), moderate (62–81%), elevated (82–99%) or outstanding (100%) [167]. Accordingly, AUC values ranged from 98.4% to 100% and from 97.8% to 99.7% when positive and negative samples were assayed with IBMP antigens using indirect ELISA and liquid microarray, respectively, as diagnostic platforms [129]. These results indicate that all four IBMP antigens have high discriminatory capability. Considering the high overall accuracy values, the IBMP antigens were used to participate in a phase II study with *T. cruzi* human positive and -negative samples from different geographic endemic regions of Brazil and other endemic countries using indirect ELISA [156–158] and liquid microarray [162]. Sensitivity, specificity and diagnostic odds ratio values were obtained that were higher than those obtained with commercial tests [156–158, 162, 168]. Cross-reactivity with *Leishmania* spp. was extremely low in patients with American cutaneous and visceral leishmaniasis [160]. In light of the negligible cross-reactivity, the authors recommend the use of IBMP antigens in regions where *T. cruzi* and *Leishmania* spp. are co-endemic [160]. In 2020, Silva et al. [163] proposed a lateral flow assay using IBMP-8.1 and IBMP-8.4 chimeric antigens for the diagnosis of CD in humans. The study showed that the assay can correctly diagnose both *T. cruzi*-positive and -negative individuals regardless of geographic origin or clinical presentation. AUC values were 100%, demonstrating an outstanding diagnostic accuracy. The study showed that the lateral flow assay based on these antigens is a promising method for screening CD [163]. In 2020, this device was licensed by the Brazilian Health Regulatory Agency to form the portfolio of diagnostic products of the Brazilian Ministry of Health for use in the Unified Health System: the TR-Chagas Bio-Manguinhos (Oswaldo Cruz Foundation, Rio de Janeiro, RJ, Brazil) [169]. Recently, all four IBMP antigens have shown promising results in a phase 3 study with more than 5000 samples from a Brazilian blood bank, especially the IBMP-8.3 and IBMP-8.4 antigens [161].

With the exception of IBMP antigens, all chimeric recombinant proteins discussed here (CP1, CP2, CP3, 2/D/E/Lo1.2, r2/D/E/Lo1.2, TcBCDE) have been evaluated only for human diagnostics. As mentioned previously, there are no commercial tests for the diagnosis of CD in dogs and cats. In 2019, a phase I study investigated the diagnostic performance of IBMP antigens in dogs [170]. AUC values ranged from 91–100%, demonstrating good diagnostic performance of these molecules also for the diagnosis of CD in dogs. Cordeiro et al. [165] reached the same conclusion by showing that IBMP-8.1 reached a maximum AUC value for both human and canine samples using an impedimetric immunosensor for rapid detection of anti-*T. cruzi* antibodies. Recently, two recombinant *T. cruzi* proteins (IBMP-8.1 and IBMP-8.4) were tested as diagnostic platforms using a rapid immunochromatographic assay (TR Chagas, Bio-Manguinhos, Rio de Janeiro, Brazil) [171]. Recombinant antigens were formatted in a rapid immunochromatographic assay using either *Staphylococcus aureus* protein A or *Streptococcus pyogenes* protein G as gold-labeled reagents to visualize the precipitin band formed between immunoglobulin (Ig) G-specific antibodies and the recombinant antigen immobilized on the nitrocellulose strip used in the assay. Protein A and protein G were based on the fact that these microbial molecules bind with different affinity and specificity to immunoglobulins of different species, including dogs. The authors found that the intensity pattern of the bands was directly proportional to the serological titer in IFAT. The sensitivity was 94% and the specificity was 91%. The agreement obtained was considered substantial by kappa analysis (84%). Of the *T. cruzi*-positive hemoculture samples, 88.9% were positive with TR-Chagas Bio-Manguinhos. The assay was efficient in detecting infections with five of the six *T. cruzi* discrete typing units (DTU; TcI, *n* = 8; TcII, *n* = 1; TcI/TcII, *n* = 2; TcIII, *n* = 2; TcIV, *n* = 1; TcIII/TcV, *n* = 6). Cross-reactions were not observed in infections with *Leishmania infantum*, *Trypanosoma rangeli*, *Trypanosoma caninum* and *Dirofilaria immitis*, but were observed in sera from dogs infected with *Crithidia mellificae*, *Anaplasma* spp. and *Erlichia* spp. However, the authors used a convenient serum panel for cross-reactivity analysis, many of which had only a single sample per disease. Therefore, further studies should be conducted to confirm or refute these results. This test provides rapid preventive measures in areas at high risk for Chagas disease occurrence in a safe, reliable, cost-effective and immediate manner without the need for more complex laboratory testing. In 2020, the diagnostic performance of a rapid test based on trypomastigote small surface antigen (TSSA) was evaluated (namely Chagas Sero K-SeT). However, low sensitivity for the diagnosis of Chagas disease in dogs was observed (28%; 16/57), indicating the need for further studies to improve test performance [172].

## Conclusion

Although the detection of anti-*T. cruzi* antibodies is possible in any mammalian species, serological tests may give discrepant results in different situations. This is mainly due to the high genetic and phenotypic intraspecific diversity of *T. cruzi* [173, 174], the selection of antigens used to sensitize the solid phase of immunoassays [133], the variable prevalence of the disease [175, 176] and the variable immune responses in *T. cruzi*-infected individuals [177]. The development of commercial diagnostic tools to detect past exposure to *T. cruzi* in dogs and cats would be useful from both veterinary and public health perspectives. Such a test should be able to detect antibodies regardless of the geographical region and the circulating DTU, with high sensitivity, specificity and accuracy, as well as with low risk of cross-reactivity (especially with *Leishmania* spp.). Furthermore, the test should be rapid (rapid diagnostic test), inexpensive and easy-to-use under field conditions (point-of-care test). Data show that the chimeric recombinant antigens combine all the necessary characteristics for a test with good applicability for epidemiological surveillance in veterinary clinical practice and in animal blood centers.

## Supplementary Information

**Additional file 1.** Preferred Reporting Items for Systematic Reviews and Meta-Analyses (PRISMA).

## Data Availability

All the data generated or analyzed during this study are included in this published article.

## Abbreviations

A: Armadillos
AST: Aspartate transferase
AUC: Area under the receiver-operator curve
C: Cats
CD: Chagas disease
CFT: Complement fixation test
D: Dogs
DAT: Direct agglutination test
DeCS: Health sciences descriptors
CK: Creatine kinase
CK-MB: Creatine kinase myocardial band
DNA: Deoxyribonucleic acid
DTU: Discrete typing units
ECG: Electrocardiogram
ELISA: Enzyme-linked immunosorbent assay
FELV: Feline leukemia virus
FIV: Feline immunodeficiency virus
FRA: Flagellar repetitive antigen
IBMP: Portuguese acronym for Biology Molecular Instituto of Paraná
IBT: Immunoblot test
IFAT: Immunofluorescence antibody test
IgG: Immunoglobulin G
IgM: Immunoglobulin M
MABA: Microplate Alamar Blue Assay
MAP: Microtubule-associated protein
PRISMA: Preferred reporting items for systematic reviews and meta-analyses
RDT: Rapid diagnostic test/immunochromatography
Scielo: Scientific electronic library online
SAPA: Shed acute phase antigen
TSSA: Mucin trypomastigote small surface antigen
WB: Western blot
WM: Wild mammals
